# Supplementary material for: Development and Validation of a Canine Health-Related Quality of Life Questionnaire and a Human–Canine Bond Questionnaire for Use in Veterinary Practice
Source: Animals (Basel). 2023 Oct 18;13(20):3255. doi: 10.3390/ani13203255 (PMC10603719; doi:10.3390/ani13203255)
Supplement: Supplementary file 1 [file animals-13-03255-s001.zip › animals-2568125-supplementary.pdf]

Supplemental Table S1. Sociodemographic Characteristics of Focus Group Participants.

**Characteristic**	**N (%)**<br>N = 20
Sex	
Female	16 (80)
Male	4 (20)
Age	
Mean age	45
Age range	27–60
Race/Ethnicity	
White/European-American	16 (80)
Black/African-American	2 (10)
Asian/Asian-American	1 (5)
Mixed	1 * (5)
Latino/Hispanic	0 (0)

* The participant self-described themselves as White and American Indian.

Supplemental Table S2. Sociodemographic Characteristics of Focus Group Participants' Dogs.

**Characteristic**	**N (%)**<br>N = 25
Age	
Mean age	7.3
Age range	1–17.75
Time spent caring for dog	
1–5 years	11 (44)
5–10 years	8 (32)
Over 10 years	6 (24)
Frequency of vet visits	
Every month	3 (12)
Every 6–8 weeks	1 (4)
Every 3–6 months	4 (24)
Twice per year	5 (20)
Once per year	9 (36)
As needed	1 (4)
Training	
Service dog training	0 (0)
Therapy dog training	1 (4)
Citizen training	1 (4)
Emotional support training	1 (4)
Health status	
Healthy	17 (68)
Unhealthy	8 (32)

Supplemental Table S3. Sociodemographic Characteristics for Cognitive Interview Participants.

**Characteristic**	**N (%)**<br>N = 16
Sex	
Female	9 (56)
Male	7 (44)
Age	
Mean age	49
Age range	20–58
Race/Ethnicity	
White/European-American	8 (50)
Black/African-American	3 (17)
Asian/Asian-American	1 (6)
Native American/Alaskan Native	1 (6)
Native Hawaiian/Pacific Islander	1 (6)
Mixed	2 * (13)
Latino/Hispanic	1 (6)
Marriage status	
Married	6 (38)
Single, not living with partner	5 (31)
Partnership/cohabitating	4 (25)
Widowed	1 (6)
Employment status	
Working full-time	9 (56)
Working part-time	3 (17)
Student	3 (17)
Retired	1 (6)
Highest level of education	
Some college	4 (25)
2-year Associate's degree	2 (13)
4-year Bachelor's degree	5 (31)
Master's degree	4 (24)
Doctoral or professional degree	1 (6)
Income level	
Less than $30,000	2 (13)
$30,000-$59,999	5 (31)
$60,000-$89,999	5 (31)
$90,000-$119,999	1 (6)
$120,000-$149,999	2 (13)
$150,000 or more	1 (6)
Children under 18 in the household	
Yes	5 (31)
No	11 (69)

* The participants self-reported themselves as Black/African-American and White/European-American, and Black/African American and Native American/Alaskan Native.
